# Effect of EGFR on SQSTM1 Expression in Malignancy and Tumor Progression of Oral Squamous Cell Carcinoma

**DOI:** 10.3390/ijms222212226

**Published:** 2021-11-12

**Authors:** Yu-Kai Tseng, Chun-Feng Chen, Chih-Wen Shu, Cheng-Hsin Lee, Yan-Ting Chou, Yi-Jing Li, Huei-Han Liou, Jiin-Tsuey Cheng, Chun-Lin Chen, Luo-Ping Ger, Pei-Feng Liu

**Affiliations:** 1 Department of Orthopedics, Kaohsiung Veterans General Hospital, Kaohsiung 81342, Taiwan; yktsengiam@vghks.gov.tw; 2 Department of Stomatology, Kaohsiung Veterans General Hospital, Kaohsiung 81342, Taiwan; johnmajor@vghks.gov.tw; 3 School of Dentistry, Kaohsiung Medical University, Kaohsiung 80708, Taiwan; 4 Department of Dental Technology, Shu-Zen Junior College of Medicine and Management, Kaohsiung 82144, Taiwan; 5 Institute of BioPharmaceutical Sciences, National Sun Yat-sen University, Kaohsiung 80424, Taiwan; cwshu@g-mail.nsysu.edu.tw; 6 Department of Biomedical Science and Environmental Biology, College of Life Science, Kaohsiung Medical University, Kaohsiung 80708, Taiwan; R980084@kmu.edu.tw (C.-H.L.); qwe781688@gmail.com (Y.-T.C.); 7 Department of Biological Sciences, National Sun Yat-sen University, Kaohsiung 80424, Taiwan; lee720127@yahoo.com.tw (Y.-J.L.); tusya@mail.nsysu.edu.tw (J.-T.C.); chunlinchen@mail.nsysu.edu.tw (C.-L.C.); 8 Department of Medical Education and Research, Kaohsiung Veterans General Hospital, Kaohsiung 81342, Taiwan; slove0726@hotmail.com (H.-H.L.); lpger0329@gmail.com (L.-P.G.); 9 Department of Medical Research, Kaohsiung Medical University Hospital, Kaohsiung 80708, Taiwan; 10 Center for Cancer Research, Kaohsiung Medical University, Kaohsiung 80708, Taiwan; 11 Institute of Biomedical Sciences, National Sun Yat-sen University, Kaohsiung 80424, Taiwan

**Keywords:** oral squamous cell carcinoma, sequestosome 1, epidermal growth factor receptor, malignancy, prognosis

## Abstract

Oral squamous cell carcinoma (OSCC) is one of the most common types of malignant tumor. Sequestosome 1 (SQSTM1) serves as an adaptor of autophagy for degrading protein aggregates. The regulation of autophagy by EGFR and its clinical impacts are indicated in various types of cancer. However, the association of EGFR and SQSTM1 in OSCC is still unknown. Our results show that the expression levels of SQSTM1 and EGFR proteins are higher in tumor tissues than in the corresponding tumor-adjacent (CTAN) tissues of OSCC patients. The expression levels of SQSTM1 were positively associated with the EGFR expression level. High co-expression of SQSTM1 and EGFR is associated with poor prognosis in OSCC patients. Moreover, SQSTM1 expression is decreased in EGFR-knockdown cells. Cell growth and invasion/migration are also decreased in cells with single/combined knockdowns of EGFR and SQSTM1 or in SQSTM1-knockdown cells without EGFR kinase inhibitor Lapatinib treatment compared to that in scrambled cells. However, cell growth and invasion/metastasis were not significantly different between the scrambled cells and SQSTM1-knockdown cells in the presence of Lapatinib. This study is the first to indicate the biological roles and clinical significance of SQSTM1 regulation by EGFR in OSCC.

## 1. Introduction

Oral squamous cell carcinoma (OSCC), a subset of head and neck squamous cell carcinoma, accounts for over 90% of fatal oral cancers worldwide [1]. According to the National Comprehensive Cancer Network (NCCN, Plymouth Meeting, PA, USA) classification, the anatomic subsites of OSCC include buccal mucosa, tongue, mucosal lip, the gums, hard palate, retromolar trigone, alveolar ridge, and floor of the mouth [2]. Among these subsites, tongue and buccal cavity cancers are relatively more common followed by lip and palate [3]. OSCC is an aggressive cancer due to its propensity for local recurrence and lymph node metastasis [4]. Surgery, radiotherapy, chemotherapy, or combinations of these treatments have been the major modalities for OSCC patients [5]. After treatment, the 5-year survival rate of OSCC patients remains at nearly 50% and has not been significantly improved in Asia over the past few years [6,7]. Thus, novel reliable biomarkers and therapeutic targets are urgently required for OSCC patients.

Autophagy is a double-edged sword in tumor progression, having both tumor-suppressive and tumor-promoting functions. In early-stage tumorigenesis, autophagy exhibits suppressive effects via inhibiting chronic inflammation or degrading potential oncogenic molecules. However, in advanced tumorigenesis, it promotes cancer cell survival under stress conditions such as hypoxia and starvation [8]. Sequestosome 1 (SQSTM1) acts as an autophagy adaptor for incorporating polyubiquitinated cytoplasmic components and organelles to the autophagosome, then fusing with lysosome for bulk degradation [9]. SQSTM1 accumulation resulting from autophagy inhibition has been detected in several cancers and may contribute to tumor progression [10]. For example, NSCLC patients with high expression of SQSTM1 have shorter survival times [11]. The high SQSTM1 expression was significantly correlated with poor prognosis in endometrial cancer [12].

Epidermal growth factor receptor (EGFR), a member of the large family of growth factor receptors, acts as an oncogenic receptor tyrosine kinase [13]. It is strongly associated with cell proliferation and malignant tumor progression [14]. The expression levels of EGFR are increased in various cancers and are associated with poor prognosis [15]. EGFR can inhibit autophagy by targeting negative regulators of autophagy, such as PI3K, AKT, and mTOR [16], contributing to chemoresistance and tumor progression [17]. So far, the link between EGFR and SQSTM1 remains poorly understood in OSCC.

This study examines the biological roles of SQSTM1 regulation by EGFR in malignancy of OSCC cells and the clinical significance of EGFR/SQSTM1 co-expression in the prognosis of OSCC patients, which might provide a potential biomarker or therapeutic target for OSCC patients.

## 2. Results

### 2.1. Comparison and Correction of EGFR and SQSTM1 Expressions in OSCC Patients

**Figure 1:**
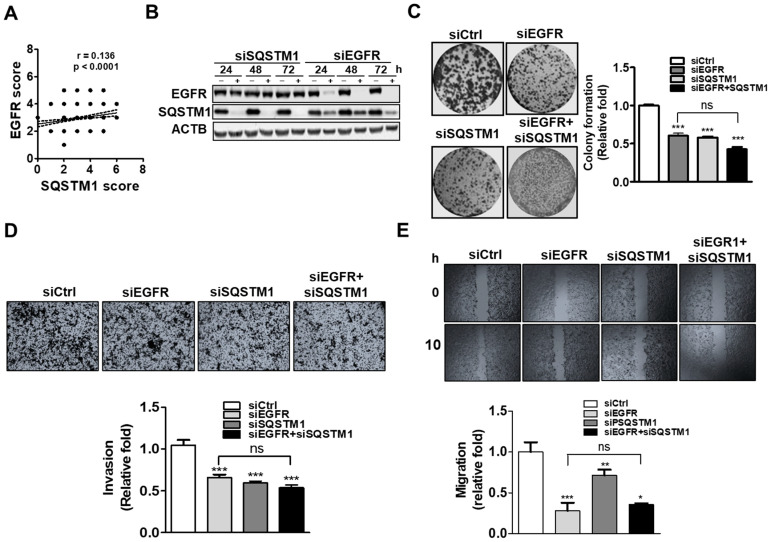
Correlation and regulation between EGFR and SQSTM1 in OSCC. (**A**) Correlation between SQSTM1 and EGFR expression in tumor tissues of OSCC patients. (**B**) Protein expression levels of EGFR and SQSTM1 in SAS cells silenced by scrambled siRNA ((−), siCtrl), SQSTM1 siRNA, or EGFR siRNA (+) for 24–72 h with Western blotting (ACTB: beta-actin as loading control). (**C**) Colony formation of SAS cells silenced by siRNA against SQSTM1-, EGFR-, or SQSTM1 + EGFR for 72 h analyzed using the clonogenic assay. (**D**) Invasion of SQSTM1-, EGFR-, or SAS cells silenced by siRNA against SQSTM1-, EGFR-, or SQSTM1 + EGFR for 72 h, evaluated using the transwell invasion assay. (**E**) Migration of SAS cells silenced by siRNA against SQSTM1-, EGFR-, or SQSTM1 + EGFR for 72 h, measured using the wound-healing assay. The mean ± SEM from three independent experiments was calculated. A two-sided *p*-value less than 0.05 was considered statistically significant. (* *p* < 0.05; ** *p* < 0.01; *** *p* < 0.001; ns: not significant).

To compare protein expression levels of EGFR and SQSTM1 between corresponding tumor-adjacent normal tissues (CTAN) and tumor tissues in OSCC patients, EGFR and SQSTM1 expressions were analyzed by immunohistochemistry (IHC) staining. The resulting protein expression levels of EGFR and SQSTM1 in tumor tissue of all OSCC patients including buccal mucosa squamous cell carcinoma (BMSCC) and tongue squamous cell carcinoma (TSCC) patients were significantly higher than those in the CTAN tissues (all *p* < 0.001, Table 1). Moreover, protein expression levels of SQSTM1 were positively correlated with that of EGFR in tumor tissues of OSCC patients (r = 0.136, *p* < 0.001; Figure 1A). These results indicate that SQSTM1 and EGFR are highly expressed and positively correlated in tumor tissues of OSCC patients.

### 2.2. Association of EGFR and SQSTM1 Expressions with Prognosis in OSCC Patients

To assess the potential value of EGFR and SQSTM1 as prognostic biomarkers for OSCC patients, we further investigated the association of EGFR and SQSTM1 expressions with prognosis including disease-specific survival (DSS) and disease-free survival (DFS). As shown in Table 2, the high expression level of SQSTM1 [adjusted hazard ratios (AHR) = 1.51, 95% confidence interval (CI ) = 1.12–2.04, *p* = 0.006] was associated with DSS in OSCC patients, especially in BMSCC patients [AHR = 1.85, 95% CI = 1.16–2.94, *p* = 0.010)]. Moreover, high co-expression of EGFR and SQSTM1 was associated with significantly poorer DSS [AHR = 1.59, 95% CI = 1.02–2.47, *p* = 0.042] in OSCC patients, especially in BMSCC patients [AHR = 2.26, 95% CI = 1.13–4.52, *p* = 0.021]. However, the co-expression of EGFR and SQSTM1 was not associated with DFS in OSCC patients (Table 3). Moreover, another cohort from the TCGA database found that oral cancer patients with high co-expression of EGFR and SQSTM1 had poor DSS (AHR = 2.53, 95% CI = 1.46–4.39, *p* = 0.001; Table 4) but not DFS. These results indicate that the high co-expression of EGFR and SQSTM1 was associated with poor prognosis in OSCC patients.

### 2.3. Regulation of SQSTM1 by EGFR and Their Roles in Cell Growth and Invasion/Migration of OSCC Cells

To verify the association of SQSTM1 and EGFR in OSCC, OSCC cells were knockdowned by siRNA or shRNA against SQSTM1 and EGFR. After knockdown, their expressions were analyzed by Western blotting (WB). SQSTM1 expression was found to be significantly decreased in EGFR-knockdown cells, indicating that SQSTM1 may be regulated by EGFR (Figure 1B). Moreover, colony formation (Figure 1C), invasion (Figure 1D), and migration (Figure 1E) of cells with siRNA against either SQSTM1 or EGFR were significantly decreased compared to that of cells knockdown by scramble siRNA (siCtrl). Knockdown both EGFR and SQSTM1 had no synergistic effects compared to knockdown EGFR alone. These results indicate that the regulation of SQSTM1 by EGFR might be required for malignant phenotypes of OSCC cells.

### 2.4. Cell Viability and Invasion/Migration of SQSTM1-Knockdowned Cells in the Presence of EGFR Kinase Inhibitor

**Figure 2:**
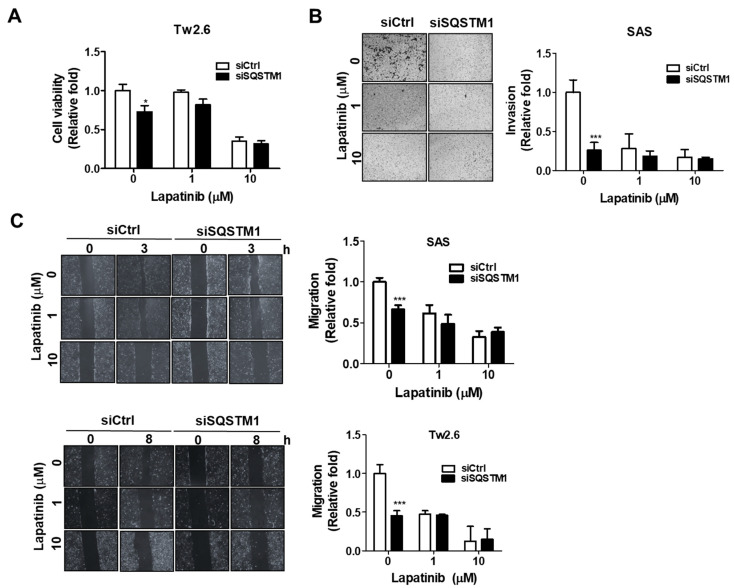
Effects of EGFR inhibitors on cell malignancy in SQSTM1-silenced OSCC cells. (**A**) Cell viability of TW2.6 cells silenced by siRNA against SQSTM1 for 48 h, then treated with Lapatinib (0–10 μM) for 24 h, measured with the CellTiter-Glo luminescent cell viability assay. (**B**) Invasion of SAS cells silenced by siRNA against SQSTM1 for 48 h, then treated with Lapatinib (0–10 μM) for 24 h, measured using the transwell invasion assay. (**C**) Migration of SAS and TW2.6 cells silenced by siRNA against SQSTM1 for 48 h, then treated with Lapatinib (0–10 μM) for 24 h, measured with the wound-healing assay. The mean ± SEM from three independent experiments was calculated. A two-sided *p*-value less than 0.05 was considered statistically significant (* *p* < 0.05; *** *p* < 0.001).

To further confirm the role of SQSTM1 regulation by EGFR in cell malignancy, malignant phenotypes of SQSTM1-knockdown OSCC cells in the absence or presence of EGFR kinase inhibitor Lapatinib were measured. Our results indicated that cell viability (Figure 2A), invasion (Figure 2B), and migration (Figure 2C) of SQSTM1-knockdown cells (siSQSTM1) were found to be significantly decreased compared to that of control cells (siCtrl) without Lapatinib treatment. Nevertheless, the cell viability of SQSTM1-knockdown cells was not significantly different from that of control cells (siCtrl) in the presence of 1–10 μM Lapatinib. The above results verified that the SQSTM1 regulation by EGFR was significantly associated with the malignancy of OSCC cells.

## 3. Discussion

Amplified or over-activated EGFR represses autophagy for growth, survival, and chemotherapy resistance in many cancers [18]. However, the molecular mechanism of autophagy regulation by EGFR remains poorly understood in cancers, especially in OSCC. This study finds that (1) The protein expression levels of EGFR and SQSTM1 in tumor tissue are significantly higher than that in normal tissue and their expression is positively correlated in OSCC patients; (2) high levels of EGFR are significantly associated with advanced pathological stage, larger tumor size, and lymph node metastasis in OSCC patients, especially in TSCC patients; (3) OSCC patients with the high co-expression of EGFR and SQSTM1 have poor DSS; (4) expression levels of SQSTM1 are significantly decreased in EGFR-knockdown OSCC cells. (5) SQSTM1 regulation by EGFR is involved in cell growth, invasion, and migration of OSCC cells. Our findings are the first to indicate the clinical significance and biological roles of SQSTM1 regulation by EGFR in OSCC.

SQSTM1 was identified as the adaptor of autophagy. The SQSTM1 protein was degraded when autophagy is induced, whereas defective autophagy could result in SQSTM1 accumulation [19]. Recent studies have reported that SQSTM1 not only acts as the adaptor of autophagy but also is an oncogenic protein that regulates several pathways for tumor progression [20]. Previous studies have also indicated that SQSTM1 can modulate autophagy for tumorigenesis and prognosis in OSCC [21] and gastric cancer [22]. SQSTM1 is also considered a predictive biomarker for drug resistance and prognosis in epithelial ovarian cancer [23]. SQSTM1 accumulation leads to chronic inflammation in liver cancer [24]. On the other hand, EGFR is involved in regulating the proliferation and survival of many types of cancer cells [14]. The abnormal activation of EGFR is significantly associated with cancer development and progression [25]. Colorectal cancer patients with high expression levels of EGFR have poor prognoses [26] and NSCLC patients with EGFR-positive expression had a higher incidence of brain metastases [27]. Interestingly, alterations in autophagic response were found in EGFR-deregulated cells and tumors [18]. The expression level of SQSTM1 positively correlates with the expression level of EGFR in breast cancer [28]. Moreover, HNSCC patients with increased SQSTM1 expression had poor response to EGFR inhibitor (cetuximab) therapy [29]. The present study also indicates the biological role and clinical significance of SQSTM1 regulation by EGFR in OSCC.

EGFR mostly functions on the cell membrane since it can traffic to the cytoplasm or other subcellular organelles [30]. Different sub-localizations of EGFR may exert different functions for cancer progression [31]. For example, cytoplasmic EGFR (EGFR-C) is associated with advanced clinicopathological features and poor prognosis in renal cell carcinoma [32] and squamous cell carcinoma of the lung [33]. This study also assessed the protein expression levels of membrane EGFR (EGFR-M) and EGFR-C in CTAN tissues and tumor tissues of OSCC patients using a semiquantitative scoring approach (Figure S1A). Both expression levels of EGFR-M and EGFR-C were higher in tumor tissues than that in CTAN tissues (all *p* < 0.001, Table S1) but only EGFR-C expression was positively correlated with SQSTM1 expression (Figure S1B). Moreover, high expression levels of EGFR-M were associated with lymph node metastasis in OSCC patients (*p* = 0.019, Table S2), especially in BMSCC patients (*p* = 0.031, Table S2). Furthermore, high expression levels of EGFR-C were associated with advanced pathological stage (*p* < 0.001, Table S3), larger tumor size (*p* = 0.006, Table S3) and lymph node metastasis (*p* = 0.012, Table S3) in OSCC patients, especially in TSCC patients. To further determine whether cytoplasmic or membrane EGFR plays a more important role in the prognosis of OSCC patients, we analyzed the association of SQSTM1/EGFR-M or EGFR-C co-expression with DSS and DFS. In DSS, the high co-expression of SQSTM1/EGFR-C was associated with poor DSS in OSCC patients (AHR = 1.57, 95% CI = 1.05–2.36, *p* = 0.030; Table S5), especially in BMSCC patients (AHR = 1.85, 95% CI = 1.16–2.94, *p* = 0.010; Table S5) but the high co-expression of SQSTM1/EGFR-M was not associated with DSS (Table S4). In DFS, neither the high co-expression of SQSTM1/EGFR-M (Table S6) nor EGFR-C (Table S7) were associated with DSS. These results indicate that EGFR-C might have more important clinical significance than EGFR-M in OSCC patients, which is consistent with the previous study indicating that amplified EGFR-C appeared to be an effective biological indicator of EGFR-driven signaling in glottic cancer [34]. Interestingly, intracellular EGFR can colocalize with Beclin 1, leading to Beclin 1 phosphorylation and inhibition of its autophagy function [17]. To further confirm how EGFR-C regulates SQSTM1, it is worth determining whether EGFR-C could bind to Beclin1 for autophagy inhibition.

## 4. Materials and Methods

### 4.1. Tissue Specimens

This study was approved by the Institutional Review Board at the Kaohsiung Veterans General Hospital (VGHKS 11-CT12–13, Kaohsiung, Taiwan) and conducted according to the guidelines of the Declaration of Helsinki. All paraffin-embedded tissues and clinicopathological data were collected from OSCC patients (*n* = 429) including BMSCC (*n* = 182) andTSCC (*n* = 247) patients between 1993 and 2006, since the survival time of OSCC patients was estimated from the time of operation to October 2012. Disease-specific survival (DSS) and disease-free survival (DFS) were calculated as in our previous study [35]. We followed up with our postoperative patients according to the oral cancer treatment guideline of VGHKS. Patients had a whole tumor image examination every six months for the first 3 years after the operation, and then every year until the fifth year. Preoperative biopsy diagnosis and post-surgery staging pathological diagnosis were routinely performed. Postoperative patients with recurrences were based on incisional biopsy diagnosis on the primary site and fine-needle aspiration on neck lymph nodes with the cooperation of imaging conformation. Pathologic TNM classification was determined in accordance with the guidelines of the 2002 American Joint Committee on Cancer (AJCC) system.

### 4.2. Tissue Microarray (TMA) Construction

A TMA block consists of cores from the tumor tissue and CTANl tissues. All TMA blocks were constructed after excluding incorrect cores and cut in 4 μm paraffin sections for further IHC analysis [35].

### 4.3. IHC

The paraffin sections cut from TMA were first dewaxed with xylene, then rehydrated by using a gradient series of alcohols, and finally washed with phosphate-buffered saline (PBS) for 5 min. Antigen retrieval was performed by immersing the section in sodium citrate (10 mM, PH 6.0) at 125 °C for 10 min. The 3% hydrogen peroxide in methanol was used to quench the endogenous peroxidase activity. Afterwards, the sections were incubated at 4 °C overnight with SQSTM1 rabbit polyclonal antibody (dilution 1:1000; Enzo Life Sciences, Farmingdale, NY, USA) and EGFR rabbit polyclonal antibody (dilution 1:50; Santa Cruz Biotechnology Inc, Santa Cruz, CA, USA). All detailed procedures for IHC were performed according to the Novolink max polymer detection system and our previous study [36]

### 4.4. IHC Scoring

The intensity and percentage of the stained positive cells were determined using the semiquantitative scoring approach and these two scores were added to obtain total scores ranging from 0 to 7. The low and high expression levels of SQSTM1 and EGFR were divided with the cutoff set at the median of score distributions. All detailed procedures for IHC scoring were achieved according to our previous study [21].

### 4.5. Cell Culture

TSCC cells (SAS) and BMSCC cells (TW2.6) were cultured in DMEM/F12 (Invitrogen-GIBCO, Carlsbad, CA, USA) containing 10% FBS, 1% L-glutamine, 100 U/mL penicillin, and 100 μg/mL streptomycin at 37 °C with 5% CO_2_.

### 4.6. WB

SDS-PAGE was used to resolve the proteins of the cell lysates. The membrane was incubated with primary antibodies at 4 °C overnight after being blocked with 5% skim milk. Next, the membrane was probed with HRP-labeled secondary antibodies, after which the ECL reagent was added. The protein expression on the membrane was detected with the ChemiDoc XRS Imaging System (Bio-Rad Laboratories, Irvine, CA, USA) and quantified.

### 4.7. Transient Transfection

The scrambled siRNA or siRNA (10 nM) against SQSTM1 or EGFR (Ambion, Austin, TX, USA) were transfected into the cells using the RNAiMAX Transfection Kit (Invitrogen Life Technologies, Carlsbad, CA, USA) for 72 h. The gene knockdown efficiency was analyzed by RT-PCR or WB [37].

### 4.8. Cell Viability

About 5–7 × 10^5^ cells/mL cultured in 96-well plates were lysed by adding 100 μL of CellTiter-Glo reagent from the CellTiter-Glo luminescent cell viability assay kit (Promega, Madison, WI, USA) for 10 min and cell viability was measured by luminescence signal in a luminometer [38].

### 4.9. Clonogenic Assay

About 500 or 1000 cells/well in 6-well plates were cultured in complete medium that was refreshed every 3 d. Afterwards, the formed cell colonies were fixed with 2% paraformaldehyde, stained with 20% ethanol containing 0.25% crystal violet for 30 min, and washed three times with PBS. The stained colonies were observed under microscopy and quantified [39].

### 4.10. Cell Invasion Assay

The 8 μm pore inserts (Greiner Bio-One, Stroud, UK) were used for transwell invasion assay. The upper side of the filter was first covered with 0.5% Matrigel ("Corning Incorporated, Corning, NY, USA, after which 8 × 10^4^ cells in 300 μL DMEM containing 1% FBS were seeded in. Complete medium was added to the bottom wells to stimulate invasion. After invasion, cells adhered to the underside of the filter were fixed in 4% formaldehyde and stained with 0.1% crystal violet. The number of migrated cells was observed and quantified [38].

### 4.11. Wound-Healing Assay

The IBIDI Culture-Inserts (IBIDI Culture-Inserts (IBIDI, Inc., Planegg, Germany) were first attached to the culture plates, then cells (in 140 µL DMEM at a density of 1.5 × 10^5^ cells/mL) were cultured in the insert overnight. Subsequently, the insert was removed, and cells were rinsed with PBS and cultured in DMEM to observe the distance between migrated cells [38].

### 4.12. Statistical Analysis

Transcriptome RNA-seq data of oral cancer patients from the TCGA database (https://cancergenome.nih.gov; accessed on 13 October 2017) were downloaded for survival analysis, which the expression levels of SQSTM1 and EGFR were dichotomized as low and high expression with the receiver operating characteristic (ROC) curve. SPSS (version 20.0, SPSS Inc., Chicago, IL, USA) was used for all statistical analysis. The Wilcoxon signed-rank test was used to compare protein expression between CTAN and tumor tissues. The impact of protein expression on survival was analyzed using the Cox proportional hazards model. A two-sided *p*-value less than 0.05 was considered statistically significant.

## 5. Conclusions

This is the first study to examine the association of SQSTM1 and EGFR as well as their biological roles/clinical significance in OSCC. Moreover, the regulation of EGFR on SQSTM1 could be both direct [40] and indirect. Most importantly, the high co-expression of EGFR and SQSTM1 is a potential prognostic biomarker or therapeutic target for OSCC patients.

## Figures and Tables

**Table 1:** The comparison of EGFR and SQSTM1expressions between corresponding tumor adjacent normal and tumor tissues in OSCC patients.

Variables	No.	Tumor Adjacent Normal		Tumor		Z	*p*-Value *
		Mean ± SD	Median	Mean ± SD	Median		
**OSCC**							
EGFR	344	2.43 ± 0.67	2.00	2.97 ± 0.79	3.00	8.998	<0.001
SQSTM1	328	1.88 ± 0.87	2.00	2.82 ± 1.09	2.00	10.500	<0.001
**BMSCC**							
EGFR	141	2.51 ± 0.70	2.00	2.99 ± 0.79	3.00	5.169	<0.001
SQSTM1	134	1.88 ± 1.00	2.00	2.88 ± 1.10	2.00	6.883	<0.001
**TSCC**							
EGFR	203	2.38 ± 0.64	2.00	2.95 ± 0.79	3.00	7.416	<0.001
SQSTM1	194	1.89 ± 0.77	2.00	2.77 ± 1.08	2.00	7.905	<0.001

Abbreviations: OSCC, oral squamous cell carcinoma; BMSCC, buccal mucosa squamous cell carcinoma; TSCC, tongue squamous cell carcinoma; SD, standard deviation. * *p*-values were estimated by Wilcoxon signed-rank test.

**Table 2:** The co-expression of EGFR and SQSTM1 in disease-specific survival of OSCC patients.

Variable		No. (%)	CHR (95% CI)	*p*-Value	AHR (95% CI)	*p*-Value *
**OSCC**						
EGFR	Low (0–3)	324 (75.5)	1.00		1.00	
	High (4–7)	105 (24.5)	1.16 (0.83–1.63)	0.392	1.02 (0.73–1.44)	0.905
SQSTM1	Low (0–2)	239 (55.7)	1.00		1.00	
	High (3–7)	190 (44.3)	1.43 (1.07–1.93)	0.017	1.51 (1.12–2.04)	0.006
EGFR (L) SQSTM1 (L)		192 (44.8)	1		1	
either		179 (41.7)	1.18 (0.88–1.59)	0.277	1.31 (0.95–1.81)	0.097
EGFR (H) SQSTM1 (H)		58 (13.2)	1.39 (0.92–2.09)	0.119	1.59 (1.02–2.47)	0.042
**BMSCC**						
EGFR	Low (0–3)	138 (75.8)	1.00		1.00	
	High (4–7)	44 (24.2)	1.35 (0.81–2.23)	0.247	1.01 (0.61–1.69)	0.962
SQSTM1	Low (0–2)	96 (52.7)	1.00		1.00	
	High (3–7)	86 (47.3)	1.72 (1.09–2.71)	0.021	1.85 (1.16–2.94)	0.010
EGFR (L) SQSTM1 (L)		74 (40.7)	1		1	
either		86 (47.3)	1.32 (0.84–2.08)	0.233	1.62 (0.97–2.70)	0.063
EGFR (H) SQSTM1 (H)		22 (12.1)	1.72 (0.93–3.20)	0.084	2.26 (1.13–4.52)	0.021
**TSCC**						
EGFR	Low (0–2)	83 (33.6)	1.00		1.00	
	High (3–7)	164 (66.4)	1.13 (0.75–1.72)	0.553	0.97 (0.64–1.47)	0.875
SQSTM1	Low (0–2)	143 (57.9)	1.00		1.00	
	High (3–7)	104 (42.1)	1.26 (0.85–1.87)	0.252	1.34 (0.90–1.98)	0.150
EGFR (L) SQSTM1 (L)		57 (23.1)	1		1	
either		112 (45.3)	0.70 (0.47–1.05)	0.083	0.81 (0.49–1.34)	0.417
EGFR (H) SQSTM1 (H)		78 (31.6)	1.48 (0.99–2.22)	0.058	1.30 (0.78–2.16)	0.316

Abbreviations: OSCC, oral squamous cell carcinoma; BMSCC, buccal mucosa squamous cell carcinoma; TSCC, tongue squamous cell carcinoma; CHR, crude hazard ratio; CI, confidence interval; AHR, adjusted hazard ratio; H, high expression; L, low expression. * *p*-value were adjusted for cell differentiation (moderate + poor vs. well) and AJCC pathological stage (stage III + IV vs. stage I + II) by multiple Cox‘s regression.

**Table 3:** The co-expression of EGFR and SQSTM1 in disease-free survival of OSCC patients.

Variable		No. (%)	CHR (95% CI)	*p*-Value	AHR (95% CI)	*p*-Value *
**OSCC**						
EGFR	Low (0–3)	324 (75.5)	1.00		1.00	
	High (4–7)	105 (24.5)	0.96 (0.68–1.36)	0.836	0.94 (0.67–1.33)	0.739
SQSTM1	Low (0–2)	239 (55.7)	1.00		1.00	
	High (3–7)	190 (44.3)	1.31 (0.98–1.76)	0.070	1.27 (0.95–1.71)	0.109
EGFR (L) SQSTM1 (L)		192 (44.8)	1		1	
either		179 (41.7)	1.20 (0.89–1.61)	0.225	1.25 (0.91–1.71)	0.165
EGFR (H) SQSTM1 (H)		58 (13.2)	1.07 (0.70–1.65)	0.750	1.20 (0.76–1.90)	0.442
**BMSCC**						
EGFR	Low (0–3)	138 (75.8)	1.00		1.00	
	High (4–7)	44 (24.2)	1.12 (0.67–1.86)	0.665	1.00 (0.60–1.68)	0.997
SQSTM1	Low (0–2)	96 (52.7)	1.00		1.00	
	High (3–7)	86 (47.3)	1.21 (0.78–1.88)	0.392	1.16 (0.74–1.81)	0.519
EGFR (L) SQSTM1 (L)		74 (40.7)	1		1	
either		86 (47.3)	1.01 (0.65–1.56)	0.979	1.09 (0.68–1.76)	0.713
EGFR (H) SQSTM1 (H)		22 (12.1)	1.37 (0.73–2.59)	0.331	1.44 (0.72–2.86)	0.300
**TSCC**						
EGFR	Low (0–2)	83 (33.6)	1.00		1.00	
	High (3–7)	164 (66.4)	1.06 (0.70–1.61)	0.782	1.04 (0.68–1.58)	0.866
SQSTM1	Low (0–2)	143 (57.9)	1.00		1.00	
	High (3–7)	104 (42.1)	1.38 (0.93–2.04)	0.112	1.35 (0.91–2.00)	0.138
EGFR (L) SQSTM1 (L)		57 (23.1)	1		1	
either		112 (45.3)	0.80 (0.54–1.20)	0.285	0.96 (0.57–1.61)	0.879
EGFR (H) SQSTM1 (H)		78 (31.6)	1.39 (0.92–2.10)	0.113	1.36 (0.80–2.31)	0.260

Abbreviations: OSCC, oral squamous cell carcinoma; BMSCC, buccal mucosa squamous cell carcinoma; TSCC, tongue squamous cell carcinoma; CHR, crude hazard ratio; CI, confidence interval; AHR, adjusted hazard ratio; H, high expression; L, low expression. * *p*-value were adjusted for cell differentiation (moderate+poor vs. well) and AJCC pathological stage (stage III + IV vs. stage I + II) by multiple Cox‘s regression.

**Table 4:** The co-expression of EGFR and SQSTM1 in survival of oral cancer patients from TCGA database.

Variable		No. (%)	CHR (95% CI)	*p*-Value	AHR (95% CI)	*p*-Value
**Overall survival**						
EGFR	Low	118 (37.8)	1.00		1.00	
	High	194 (62.2)	1.44 (1.01–2.05)	0.047 ^a^	1.47 (1.01–2.16)	0.047 ^b^
SQSTM1	Low	171 (54.8)	1.00		1.00	
	High	141 (45.2)	1.61 (1.15–2.24)	0.005 ^a^	1.44 (1.01–2.05)	0.044 ^b^
EGFR(L), SQSTM1 (L)		59 (18.9)	1.00		1.00	
EGFR (H), SQSTM1 (L)		112 (35.9)	0.88 (0.62–1.24)	0.460 ^a^	1.58 (0.91–2.73)	0.101 ^c^
EGFR (L), SQSTM1 (H)		59 (18.9)	1.02 (0.67–1.57)	0.915 ^a^	1.78 (0.96–3.29)	0.067 ^c^
EGFR (H), SQSTM1 (H)		82 (26.3)	1.71 (1.21–2.42)	0.002 ^a^	2.53 (1.46–4.39)	0.001^c^
**Disease** **-free survival**						
EGFR	Low	239 (91.2)	1.00		1.00	
	High	23 (8.8)	1.44 (0.69–3.02)	0.336 ^a^	1.48 (0.70–3.13)	0.301 ^b^
SQSTM1	Low	146 (55.7)	1.00		1.00	
	High	116 (44.3)	1.31 (0.80–2.14)	0.285 ^a^	1.15 (0.69–1.94)	0.587 ^b^
EGFR (L), SQSTM1 (L)		129 (49.2)	1.00		1.00	
EGFR (H), SQSTM1 (L)		17 (6.5)	1.85 (0.84–4.06)	0.126 ^a^	2.28 (0.98–5.28)	0.056 ^c^
EGFR (L), SQSTM1 (H)		110 (42.0)	1.38 (0.84–2.26)	0.202 ^a^	1.55 (0.91–2.63)	0.106 ^c^
EGFR (H), SQSTM1 (H)		6 (2.3)	0.55 (0.08–3.99)	0.557 ^a^	0.73 (0.10–5.36)	0.753 ^c^

Abbreviations: CHR, crude hazard ratio; CI, confidence interval; AHR, adjusted hazard ratio; H, high expression; L, low expression. ^a^ *p* values were estimated by Cox’s regression. ^b^ *p* values were adjusted for cell differentiation (moderate+poor vs. well) and AJCC pathological stage (stage III + IV vs. stage I + II) by multivariate Cox’s regression. ^c^ *p* values were estimated by multivariate Cox’s regression.

## Data Availability

Not applicable.

## Supplementary Materials

The following are available online at https://www.mdpi.com/article/10.3390/ijms222212226/s1.
