# Supplementary material for: Effect of EGFR on SQSTM1 Expression in Malignancy and Tumor Progression of Oral Squamous Cell Carcinoma
Source: Int J Mol Sci. 2021 Nov 12;22(22):12226. doi: 10.3390/ijms222212226 (PMC8625971; doi:10.3390/ijms222212226)
Supplement: Supplementary file 1 [file ijms-22-12226-s001.zip › ijms-1433252-supplementary.pdf]

## Supplementary materials

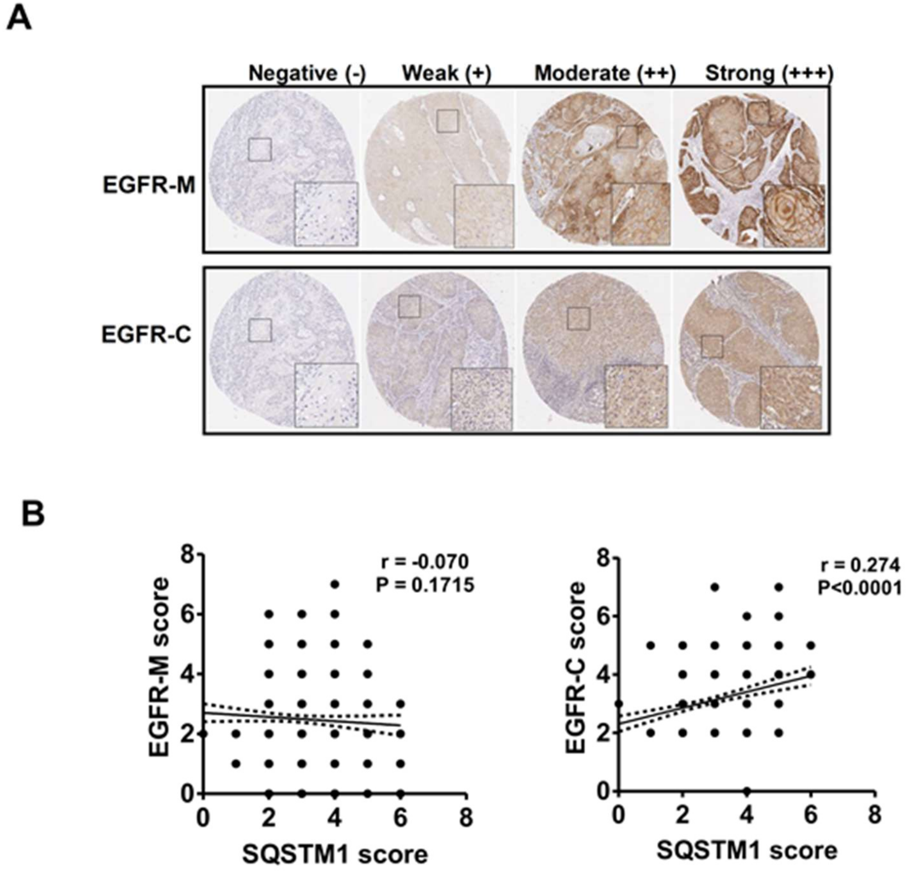


**Figure S1.** Expressions of EGFR-M and EGFR-C and their correlation with SQSTM1 in tumor tissues of OSCC patients. **(A)** Representative IHC images showing intensity scores of EGFR-M and EGFR-C staining as negative (-), weak (+), moderate (++), and strong (+++). **(B)** The correlation of EGFR-M and EGFR-C with SQSTM1 by the Pearson correlation analysis.

**Table S1.** The comparison of EGFR-M and EGFR-C expressions between corresponding tumor adjacent normal and normal tissues in OSCC patients.

| Variables | No. | Tumor adjacent normal | | Tumor | | Z | *p*-value* |
|---|---|---|---|---|---|---|---|
| | | Mean±SD | Median | Mean±SD | Median | | |
| **OSCC** | | | | | | | |
| EGFR-M | 348 | 1.48± 1.22 | 2.00 | 2.62± 1.16 | 3.00 | 10.542 | <0.001 |
| EGFR-C | 348 | 2.51± 0.86 | 2.00 | 3.08± 1.10 | 3.00 | 8.296 | <0.001 |
| **BMSCC** | | | | | | | |
| EGFR-M | 144 | 1.55± 1.27 | 2.00 | 2.54± 1.06 | 2.00 | 6.547 | <0.001 |
| EGFR-C | 144 | 2.54± 0.95 | 2.00 | 3.06± 1.07 | 3.00 | 4.994 | <0.001 |
| **TSCC** | | | | | | | |
| EGFR-M | 204 | 1.44± 1.17 | 2.00 | 2.68± 1.22 | 3.00 | 8.235 | <0.001 |
| EGFR-C | 204 | 2.49± 0.80 | 2.00 | 3.09± 1.12 | 3.00 | 6.618 | <0.001 |

*Abbreviations: OSCC, oral squamous cell carcinoma; BMSCC, buccal mucosa squamous cell carcinoma; TSCC, tongue squamous cell carcinoma; SD, standard deviation.; M, membrane; C, cytoplasmic.*

**p-valueswere estimated by Wilcoxon signed-rank test.*

**Table S2.** The association of EGFR-M expression with clinicopathologic outcomes in OSCC patients.

| Variable | **BMSCC** (n=181) | | | | **TSCC** (n=244) | | | | **OSCC** (n=425) | | | |
|---|---|---|---|---|---|---|---|---|---|---|---|---|
| | % | Mean±SD | Median | *p*-value | % | Mean±SD | Median | *p*-value | % | Mean±SD | Median | *p*-value |
| **Sex** | | | | | | | | | | | | |
| Female | 2.2 | 1.50 ± 0.58 | 1.50 | 0.079* | 11.9 | 2.34 ± 1.29 | 3.00 | 0.278* | 7.8 | 2.24 ± 1.25 | 2.00 | 0.162* |
| Male | 97.8 | 2.45 ± 1.07 | 2.00 | | 88.1 | 2.61 ± 1.22 | 2.00 | | 92.2 | 2.54 ± 1.16 | 2.00 | |
| **Age, y** | | | | | | | | | | | | |
| ≦50 | 44.2 | 2.50 ± 1.09 | 2.00 | 0.442* | 51.6 | 2.52 ± 1.20 | 2.00 | 0.479* | 48.5 | 2.51 ± 1.15 | 2.00 | 0.990* |
| ＞50 | 55.8 | 2.38 ± 1.06 | 2.00 | | 48.4 | 2.64 ± 1.27 | 3.00 | | 51.5 | 2.52 ± 1.18 | 2.00 | |
| **Cell differentiation** | | | | | | | | | | | | |
| Well | 26.0 | 2.60 ± 1.06 | 2.00 | 0.176† | 10.7 | 2.85 ± 1.08 | 3.00 | 0.164† | 17.2 | 2.68 ± 1.07 | 3.00 | 0.052† |
| Moderate | 69.1 | 2.41 ± 1.05 | 2.00 | | 82.4 | 2.58 ± 1.23 | 3.00 | | 76.7 | 2.52 ± 1.16 | 2.00 | |
| Poor | 5.0 | 1.89 ± 1.36 | 2.00 | | 7.0 | 2.12 ± 1.41 | 2.00 | | 6.1 | 2.04 ± 1.37 | 2.00 | |
| **AJCC pathological stage** | | | | | | | | | | | | |
| I, II | 61.3 | 2.51 ± 0.96 | 2.00 | 0.192* | 68.9 | 2.65 ± 1.27 | 3.00 | 0.181* | 65.6 | 2.60 ± 1.16 | 3.00 | 0.051* |
| III, IV | 38.7 | 2.30 ± 1.22 | 2.00 | | 31.1 | 2.42 ± 1.12 | 2.00 | | 34.4 | 2.36 ± 1.17 | 2.00 | |
| **T classification** | | | | | | | | | | | | |
| T1, T2 | 75.7 | 2.47 ± 1.09 | 2.00 | 0.336* | 79.5 | 2.59 ± 1.30 | 3.00 | 0.911‡ | 77.9 | 2.54 ± 1.21 | 2.00 | 0.398* |
| T3, T4 | 24.3 | 2.30 ± 1.02 | 2.00 | | 20.5 | 2.54 ± 0.93 | 2.00 | | 22.1 | 2.43 ± 0.98 | 2.00 | |
| **N classification** | | | | | | | | | | | | |
| N0 | 75.1 | 2.53 ± 0.97 | 2.00 | 0.031* | 79.9 | 2.63 ± 1.24 | 3.00 | 0.227* | 77.9 | 2.54 ± 1.21 | 2.00 | 0.019* |
| N1, N2 | 24.9 | 2.13 ± 1.31 | 2.00 | | 20.1 | 2.39 ± 1.19 | 2.00 | | 22.1 | 2.43 ± 0.98 | 2.00 | |

*Abbreviations: OSCC, oral squamous cell carcinoma; BMSCC, buccal mucosa squamous cell carcinoma; TSCC, tongue squamous cell carcinoma; AJCC, American Joint Committee on Cancer.*

* *p values were estimated by student's t-test.*

† *p values were estimated by one-way ANOVA test.*

‡ *p values was estimated by Mann-Whitney U test.*

**Table S3.** The association of EGFR-C expression with clinicopathologic outcomes in OSCC patients.

| Variable | **BMSCC** (n=181) | | | | **TSCC** (n=244) | | | | **OSCC** (n=425) | | | |
|---|---|---|---|---|---|---|---|---|---|---|---|---|
| | % | Mean±SD | Median | *p*-value | % | Mean±SD | Median | *p*-value | % | Mean±SD | Median | *p*-value |
| **Sex** | | | | | | | | | | | | |
| Female | 2.2 | 2.50 ± 0.58 | 2.50 | 0.308* | 11.9 | 3.03 ± 0.98 | 3.00 | 0.693* | 7.8 | 2.97 ± 0.95 | 3.00 | 0.566* |
| Male | 97.8 | 3.03 ± 1.04 | 3.00 | | 88.1 | 3.12 ± 1.12 | 3.00 | | 92.2 | 3.08 ± 1.08 | 3.00 | |
| **Age, y** | | | | | | | | | | | | |
| ≦50 | 44.2 | 3.11 ± 1.04 | 3.00 | 0.296* | 51.6 | 3.15 ± 1.09 | 3.00 | 0.558* | 48.5 | 3.14 ± 1.07 | 3.00 | 0.241* |
| ＞50 | 55.8 | 2.95 ± 1.02 | 3.00 | | 48.4 | 3.07 ± 1.12 | 3.00 | | 51.5 | 3.01 ± 1.08 | 3.00 | |
| **Cell differentiation** | | | | | | | | | | | | |
| Well | 26.0 | 2.94 ± 0.87 | 3.00 | 0.745‡ | 10.7 | 3.00 ± 1.17 | 2.00 | 0.209‡ | 17.2 | 2.96 ± 0.98 | 3.00 | 0.162‡ |
| Moderate | 69.1 | 3.02 ± 1.04 | 3.00 | | 82.4 | 3.08 ± 1.06 | 3.00 | | 76.7 | 3.06 ± 1.05 | 3.00 | |
| Poor | 5.0 | 3.44 ± 1.59 | 3.00 | | 7.0 | 3.65 ± 1.41 | 4.00 | | 6.1 | 3.58 ± 1.45 | 4.00 | |
| **AJCC pathological stage** | | | | | | | | | | | | |
| I, II | 61.3 | 2.94 ± 0.93 | 3.00 | 0.352† | 68.9 | 2.93 ± 1.05 | 3.00 | <0.001* | 65.6 | 2.93 ± 1.00 | 3.00 | <0.001† |
| III, IV | 38.7 | 3.16 ± 1.18 | 3.00 | | 31.1 | 3.51 ± 1.11 | 4.00 | | 34.4 | 3.34 ± 1.15 | 3.00 | |
| **T classification** | | | | | | | | | | | | |
| T1, T2 | 75.7 | 2.99 ± 1.02 | 3.00 | 0.400* | 79.5 | 3.01 ± 1.10 | 3.00 | 0.003* | 77.9 | 3.00 ± 1.07 | 3.00 | 0.006* |
| T3, T4 | 24.3 | 3.14 ± 1.07 | 3.00 | | 20.5 | 3.52 ± 1.01 | 4.00 | | 22.1 | 3.34 ± 1.05 | 3.00 | |
| **N classification** | | | | | | | | | | | | |
| N0 | 75.1 | 2.95 ± 0.93 | 3.00 | 0.312† | 79.9 | 3.02 ± 1.06 | 3.00 | 0.013† | 77.9 | 2.99 ± 1.01 | 3.00 | 0.012† |
| N1, N2 | 24.9 | 3.24 ± 1.28 | 3.00 | | 20.1 | 3.49 ± 1.21 | 3.00 | | 22.1 | 3.37 ± 1.24 | 3.00 | |

*Abbreviations: OSCC, oral squamous cell carcinoma; BMSCC, buccal mucosa squamous cell carcinoma; TSCC, tongue squamous cell carcinoma; AJCC, American Joint Committee on Cancer.*

*p values were estimated by student's t-test.*

† *p values was estimated by Mann-Whitney U test.*

‡ *p values were estimated by Kruskal-Wallis one-way ANOVA test.*

| **Table S4.** The co-expression of EGFR-M and SQSTM1 in disease-specific survival of OSCC patients. | | | | | | |
|---|---|---|---|---|---|---|
| Variable | | No. (%) | CHR (95% CI) | *p*-value | AHR (95% CI) | *p*-value * |
| **OSCC** | | | | | | |
| EGFR-M | Low (0-3) | 363 (84.6) | 1.00 | | 1.00 | |
| | High (4-7) | 66 (15.4) | 0.94 (0.61-1.43) | 0.763 | 1.12 (0.73-1.72) | 0.591 |
| SQSTM1 | Low (0-2) | 239 (55.7) | 1.00 | | 1.00 | |
| | High (3-7) | 190 (44.3) | 1.43 (1.07-1.93) | **0.017** | 1.51 (1.12-2.04) | 0.006 |
| EGFR-M (L) SQSTM1 (L) | | 200 (46.6) | 1 | | 1 | |
| either | | 202 (47.1) | 1.25 (0.92-1.68) | 0.143 | 1.30 (0.96-1.77) | 0.093 |
| EGFR-M (H) SQSTM1 (H) | | 27 (6.3) | 1.25 (0.70-2.24) | 0.460 | 1.43 (0.78-2.62) | 0.255 |
| **BMSCC** | | | | | | |
| EGFR-M | Low (0-3) | 157 (86.3) | 1.00 | | 1.00 | |
| | High (4-7) | 25 (13.7) | 0.85 (0.42-1.70) | 0.642 | 0.95 (0.47-1.93) | 0.894 |
| SQSTM1 | Low (0-3) | 96 (52.7) | 1.00 | | 1.00 | |
| | High (4-7) | 86 (47.3) | 1.72 (1.09-2.71) | 0.021 | 1.85 (1.16-2.94) | 0.010 |
| EGFR-M (L) SQSTM1 (L) | | 78 (42.9) | 1 | | 1 | |
| either | | 97 (53.3) | 1.81 (1.14-2.90) | 0.013 | 1.80 (1.11-2.90) | 0.017 |
| EGFR-M (H) SQSTM1 (H) | | 7 (3.8) | 0.63 (0.15-2.55) | 0.514 | 0.88 (0.21-3.70) | 0.858 |
| **TSCC** | | | | | | |
| EGFR-M | Low (0-2) | 125 (50.6) | 1.00 | | 1.00 | |
| | High (3-7) | 122 (49.4) | 0.88 (0.59-1.30) | 0.517 | 1.11 (0.75-1.66) | 0.598 |
| SQSTM1 | Low (0-2) | 143 (57.9) | 1.00 | | 1.00 | |
| | High (3-7) | 104 (42.1) | 1.26 (0.85-1.87) | 0.252 | 1.34 (0.90-1.98) | 0.150 |
| EGFR-M (L) SQSTM1 (L) | | 73 (29.6) | 1 | | 1 | |
| either | | 122 (49.4) | 0.80 (0.54-1.18) | 0.257 | 0.84 (0.54-1.32) | 0.458 |
| EGFR-M (H) SQSTM1 (H) | | 52 (21.1) | 1.28 (0.80-2.03) | 0.305 | 1.15 (0.67-1.97) | 0.610 |

*Abbreviations: OSCC, oral squamous cell carcinoma; BMSCC, buccal mucosa squamous cell carcinoma; TSCC, tongue squamous cell carcinoma; CHR, crude hazard ratio; CI, confidence interval; AHR, adjusted hazard ratio; H, high expression; L, low expression; M, membrane.*

**p-value were adjusted for cell differentiation (moderate+poor vs. well) and AJCC pathological stage (stage III+IV vs stage I+II) by multiple Cox's regression.*

**Table S5.** The co-expression of EGFR-C and SQSTM1 in disease-specific survival of OSCC patients.

| Variable | | No. (%) | CHR (95% CI) | *p*-value | AHR (95% CI) | *p*-value * |
|---|---|---|---|---|---|---|
| **OSCC** | | | | | | |
| EGFR-C | Low (0-3) | 285 (75.5) | 1.00 | | 1.00 | |
| | High (4-7) | 144 (24.5) | 1.21 (0.89-1.64) | 0.230 | 1.06 (0.78-1.44) | 0.718 |
| SQSTM1 | Low (0-2) | 239 (55.7) | 1.00 | | 1.00 | |
| | High (3-7) | 190 (44.3) | 1.43 (1.07-1.93) | 0.017 | 1.51 (1.12-2.04) | 0.006 |
| EGFR-C (L) SQSTM1 (L) | | 174 (40.6) | 1 | | 1 | |
| either | | 176 (41.0) | 1.21 (0.90-1.63) | 0.217 | 1.40 (1.00-1.95) | 0.051 |
| EGFR-C (H) SQSTM1 (H) | | 79 (18.4) | 1.32 (0.92-1.90) | 0.130 | 1.57 (1.05-2.36) | 0.030 |
| **BMSCC** | | | | | | |
| EGFR-C | Low (0-3) | 132 (72.5) | 1.00 | | 1.00 | |
| | High (4-7) | 50 (27.5) | 1.37 (0.84-2.21) | 0.204 | 1.19 (0.73-1.92) | 0.492 |
| SQSTM1 | Low (0-2) | 96 (52.7) | 1.00 | | 1.00 | |
| | High (3-7) | 86 (47.3) | 1.72 (1.09-2.71) | 0.021 | 1.85 (1.16-2.94) | 0.010 |
| | | | | | | |
| EGFR-C (L) SQSTM1 (L) | | 76 (41.8) | 1 | | 1 | |
| either | | 76 (41.8) | 1.15 (0.73-1.81) | 0.552 | 1.46 (0.87-2.46) | 0.152 |
| EGFR-C (H) SQSTM1 (H) | | 30 (16.5) | 1.74 (1.01-2.99) | 0.045 | 2.12 (1.15-3.94) | 0.017 |
| **TSCC** | | | | | | |
| EGFR-C | Low (0-2) | 99 (40.1) | 1.00 | | 1.00 | |
| | High (3-7) | 148 (59.9) | 0.97 (0.65-1.44) | 0.880 | 0.72 (0.48-1.08) | 0.111 |
| SQSTM1 | Low (0-2) | 143 (57.9) | 1.00 | | 1.00 | |
| | High (3-7) | 104 (42.1) | 1.26 (0.85-1.87) | 0.252 | 1.34 (0.90-1.98) | 0.150 |
| EGFR-C (L) SQSTM1 (L) | | 68 (27.5) | 1 | | 1 | |
| either | | 106 (42.9) | 1.07 (0.72-1.58) | 0.754 | 1.16 (0.72-1.87) | 0.543 |
| EGFR-C (H) SQSTM1 (H) | | 73 (29.6) | 1.08 (0.71-1.66) | 0.713 | 1.19 (0.70-1.99) | 0.523 |

*Abbreviations: OSCC, oral squamous cell carcinoma; BMSCC, buccal mucosa squamous cell carcinoma; TSCC, tongue squamous cell carcinoma; CHR, crude hazard ratio; CI, confidence interval; AHR, adjusted hazard ratio; H, high expression; L, low expression; C, cytoplasmic.*

**p-value were adjusted for cell differentiation (moderate+poor vs. well) and AJCC pathological stage (stage III+IV vs stage I+II) by multiple Cox's regression.*

**Table S6.** The co-expression of EGFR-M and SQSTM1 in disease-free survival of OSCC patients.

| Variable | | No. (%) | CHR (95% CI) | *p*-value | AHR (95% CI) | *p*-value * |
|---|---|---|---|---|---|---|
| **OSCC** | | | | | | |
| EGFR-M | Low (0-3) | 363 (84.6) | 1.00 | | 1.00 | |
| | High (4-7) | 66 (15.4) | 0.94 (0.63-1.42) | 0.776 | 1.05 (0.69-1.58) | 0.825 |
| SQSTM1 | Low (0-2) | 239 (55.7) | 1.00 | | 1.00 | |
| | High (3-7) | 190 (44.3) | 1.31 (0.98-1.76) | 0.070 | 1.27 (0.95-1.71) | 0.109 |
| EGFR-M (L) SQSTM1 (L) | | 200 (46.6) | 1 | | 1 | |
| either | | 202 (47.1) | 1.28 (0.95-1.72) | 0.100 | 1.30 (0.96-1.76) | 0.093 |
| EGFR-M (H) SQSTM1 (H) | | 27 (6.3) | 0.98 (0.53-1.81) | 0.956 | 1.12 (0.60-2.12) | 0.717 |
| **BMSCC** | | | | | | |
| EGFR-M | Low (0-3) | 157 (86.3) | 1.00 | | 1.00 | |
| | High (4-7) | 25 (13.7) | 1.05 (0.57-1.94) | 0.880 | 1.19 (0.64-2.22) | 0.585 |
| SQSTM1 | Low (0-2) | 96 (52.7) | 1.00 | | 1.00 | |
| | High (4-7) | 86 (47.3) | 1.21 (0.78-1.88) | 0.392 | 1.16 (0.74-1.81) | 0.519 |
| | | | | | | |
| EGFR-M (L) SQSTM1 (L) | | 114 (62.6) | 1 | | 1 | |
| either | | 62 (34.1) | 1.49 (0.95-2.34) | 0.079 | 1.44 (0.91-2.28) | 0.117 |
| EGFR-M (H) SQSTM1 (H) | | 6 (3.3) | 0.51 (0.13-2.09) | 0.353 | 0.63 (0.15-2.65) | 0.531 |
| **TSCC** | | | | | | |
| EGFR-M | Low (0-2) | 125 (50.6) | 1.00 | | 1.00 | |
| | High (3-7) | 122 (49.4) | 0.87 (0.58-1.29) | 0.479 | 0.92 (0.62-1.37) | 0.680 |
| SQSTM1 | Low (0-2) | 143 (57.9) | 1.00 | | 1.00 | |
| | High (3-7) | 104 (42.1) | 1.38 (0.93-2.04) | 0.112 | 1.35 (0.91-2.00) | 0.138 |
| EGFR-M (L) SQSTM1 (L) | | 73 (29.6) | 1 | | 1 | |
| either | | 122 (49.4) | 0.62 (0.42-0.93) | 0.021 | 0.69 (0.43-1.09) | 0.112 |
| EGFR (H) SQSTM1 (H) | | 52 (21.1) | 1.57 (1.01-2.45) | 0.046 | 1.25 (0.75-2.09) | 0.389 |

*Abbreviations: OSCC, oral squamous cell carcinoma; BMSCC, buccal mucosa squamous cell carcinoma; TSCC, tongue squamous cell carcinoma; CHR, crude hazard ratio; CI, confidence interval; AHR, adjusted hazard ratio; H, high expression; L, low expression; M, membrane.*

**p-value were adjusted for cell differentiation(moderate+poor vs. well) and AJCC pathological stage (stage III+IV vs stage I+II) by multiple Cox's regression.*

| **Table S7.** The co-expression of EGFR-C and SQSTM1 in disease-free survival of OSCC patients. | | | | | | |
|---|---|---|---|---|---|---|
| Variable | | No. (%) | CHR (95% CI) | *p*-value | AHR (95% CI) | *p*-value * |
| **OSCC** | | | | | | |
| EGFR-C | Low (0-3) | 285 (75.5) | 1.00 | | 1.00 | |
| | High (4-7) | 144 (24.5) | 0.90 (0.65-1.23) | 0.509 | 0.87 (0.63-1.20) | 0.383 |
| SQSTM1 | Low (0-2) | 239 (55.7) | 1.00 | | 1.00 | |
| | High (3-7) | 190 (44.3) | 1.31 (0.98-1.76) | 0.070 | 1.27 (0.95-1.71) | 0.109 |
| EGFR-C (L) SQSTM1 (L) | | 174 (40.6) | 1 | | 1 | |
| either | | 176 (41.0) | 1.16 (0.86-1.56) | 0.330 | 1.20 (0.87-1.66) | 0.266 |
| EGFR-C (H) SQSTM1 (H) | | 79 (18.4) | 1.03 (0.70-1.51) | 0.887 | 1.13 (0.74-1.72) | 0.571 |
| **BMSCC** | | | | | | |
| EGFR-C | Low (0-3) | 132 (72.5) | 1.00 | | 1.00 | |
| | High (4-7) | 50 (27.5) | 0.68 (0.40-1.17) | 0.163 | 0.65 (0.38-1.12) | 0.122 |
| SQSTM1 | Low (0-2) | 96 (52.7) | 1.00 | | 1.00 | |
| | High (3-7) | 86 (47.3) | 1.21 (0.78-1.88) | 0.392 | 1.16 (0.74-1.81) | 0.519 |
| EGFR-C (L) SQSTM1 (L) | | 76 (41.8) | 1 | | 1 | |
| either | | 76 (41.8) | 0.95 (0.61-1.47) | 0.802 | 0.93 (0.58-1.49) | 0.754 |
| EGFR-C (H) SQSTM1 (H) | | 30 (16.5) | 0.97 (0.52-1.78) | 0.909 | 0.93 (0.48-1.79) | 0.826 |
| **TSCC** | | | | | | |
| EGFR-C | Low (0-2) | 99 (40.1) | 1.00 | | 1.00 | |
| | High (3-7) | 148 (59.9) | 0.98 (0.66-1.46) | 0.919 | 0.91 (0.60-1.37) | 0.638 |
| SQSTM1 | Low (0-2) | 143 (57.9) | 1.00 | | 1.00 | |
| | High (3-7) | 104 (42.1) | 1.38 (0.93-2.04) | 0.112 | 1.35 (0.91-2.00) | 0.138 |
| EGFR-C (L) SQSTM1 (L) | | 68 (27.5) | 1 | | 1 | |
| either | | 106 (42.9) | 1.03 (0.69-1.53) | 0.900 | 1.17 (0.72-1.92) | 0.523 |
| EGFR-C (H) SQSTM1 (H) | | 73 (29.6) | 1.18 (0.77-1.80) | 0.458 | 1.30 (0.77-2.20) | 0.334 |

*Abbreviations: OSCC, oral squamous cell carcinoma; BMSCC, buccal mucosa squamous cell carcinoma; TSCC, tongue squamous cell carcinoma; CHR, crude hazard ratio; CI, confidence interval; AHR, adjusted hazard ratio; H, high expression; L, low expression; C, cytoplasmic.*

**p-value were adjusted for cell differentiation(moderate+poor vs. well) and AJCC pathological stage (stage III+IV vs stage I+II) by multiple Cox's regression.*
